# Thermoelectric effect and its dependence on molecular length and sequence in single DNA molecules

**DOI:** 10.1038/ncomms11294

**Published:** 2016-04-15

**Authors:** Yueqi Li, Limin Xiang, Julio L. Palma, Yoshihiro Asai, Nongjian Tao

**Affiliations:** 1 Center for Bioelectronics and Biosensors, Biodesign Institute, Arizona State University, Tempe, Arizona 85287-5801, USA; 2 School of Molecular Sciences, Arizona State University, Tempe, Arizona 85287-5801, USA; 3 Research Center for Computational Design of Advanced Functional Materials (CD-FMat), National Institute of Advanced Industrial Science and Technology (AIST), Central 2, Umezono 1-1-1, Tsukuba, Ibaraki 305-8568, Japan; 4 School of Chemistry and Chemical Engineering, Nanjing University, Nanjing, Jiangsu 210093 China

## Abstract

Studying the thermoelectric effect in DNA is important for unravelling charge transport mechanisms and for developing relevant applications of DNA molecules. Here we report a study of the thermoelectric effect in single DNA molecules. By varying the molecular length and sequence, we tune the charge transport in DNA to either a hopping- or tunnelling-dominated regimes. The thermoelectric effect is small and insensitive to the molecular length in the hopping regime. In contrast, the thermoelectric effect is large and sensitive to the length in the tunnelling regime. These findings indicate that one may control the thermoelectric effect in DNA by varying its sequence and length. We describe the experimental results in terms of hopping and tunnelling charge transport models.

## Abstract (web summary)

Understanding the thermoelectric effect of materials and molecules is important for understanding their charge transport properties. Here, the authors study the thermoelectric effect in single DNA molecules and show that the charge carrier properties are dependent on both the DNA length and sequence.

## Graphical abstract

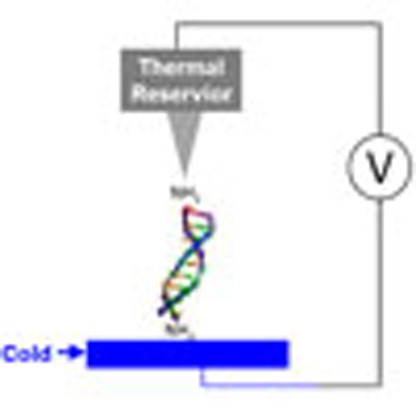

The thermoelectric effect is a basic property of materials and has found applications in energy conversion, temperature sensing and regulation. This important effect in single molecules is expected to be distinctly different from that in bulk materials^1^. In addition to potential applications, studying the thermoelectric effect in single molecules helps assess the molecular orbital level alignment, understand the energy conversion mechanism associated with charge transport and determine whether electrons or holes are responsible for the charge transport. The work by Reddy *et al*.^2^ has stimulated many efforts in investigating the thermoelectric effect in molecules^3^,^4^,^5^,^6^. However, to date, research has been limited to the regime where the Fermi levels of the electrodes are in the highest occupied molecular orbital (HOMO)–lowest unoccupied molecular orbital (LUMO) gaps of the molecules, and electrons or holes transport through molecules via coherent tunnelling, a quantum mechanical process with a characteristic exponential dependence of the molecular resistance with length.

Here we report a study of the thermoelectric effect in double-stranded DNA (dsDNA) molecules. By selecting different DNA sequences, we study the thermoelectric effect not only in the tunnelling regime but also in the hopping regime, in which electrons or holes hop along a molecule from one end to another sequentially via multiple steps, leading to a linear dependence of the molecular resistance with length. The work demonstrates that DNA thermoelectricity can be tuned by its sequences and length, which provides new insights into the thermoelectric effect in single molecules, and basic knowledge for potential applications with programmable DNA nanostructures, a field that has been advancing rapidly in recent years^7^.

## Results

### dsDNA sequences

**Figure 1:**
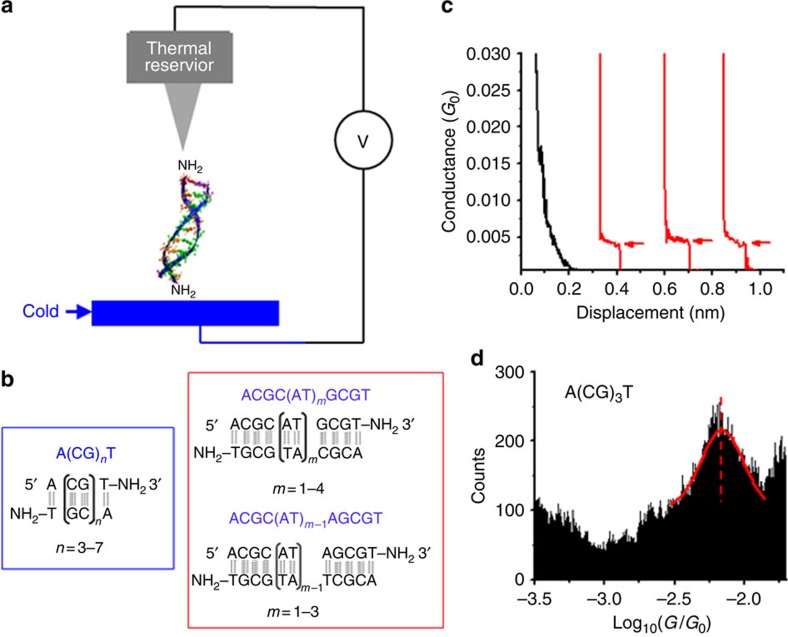
Thermoelectric and conductance measurements of DNA. (**a**) Conductance and thermoelectric effect of a DNA molecule bridged between STM tip (kept at 295 K) and substrate (cold). (**b**) Two families of DNA sequences are studied in this work, denoted as (1) A(CG)_*n*_T, (2) ACGC(**AT**)_*m*_GCGT and ACGC(**AT**)_*m*−1_**A**GCGT (see Table 1 for a full list). (**c**) Conductance traces without (black) and with (red) dsDNA A(CG)_3_T, where plateaus are marked with red arrows. (**d**) Conductance histogram of A(CG)_3_T, where the solid red curve is Gaussian fit to the conductance peak. Note: *G*_0_=2*e*^2^/*h*=77.48 μS.

To study the thermoelectric effect in the hopping regime, we selected DNA molecules with sequences of 5′-A(CG)_*n*_T-3′ (*n*=3, 4, 5, 6 and 7; Fig. 1b), where A, C, G and T denote the four DNA bases, adenine, cytosine, guanine and thymine, respectively. Note that this sequence notation shows only one strand of the dsDNA, and the second strand has the complementary sequence. The complete sequences of all the dsDNA studied in this work are listed in Table 1. Previous studies have shown that charge transport in GC sequences is dominated by hopping of holes along the molecules with G as hopping sites^8^,^9^,^10^,^11^,^12^,^13^. To investigate the thermoelectric effect in the tunnelling regime, we studied DNA sequences, 5′-ACGC(**AT**)_*m*_GCGT-3′, where *m*=1, 2, 3 and 4, and 5′-ACGC(**AT**)_*m*−1_**A**GCGT-3′, where *m*=1, 2 and 3 (Fig. 1b; Table 1). The insertion of a block of AT bases in the middle of the GC sequences introduces a tunnelling barrier into the DNA molecules, as shown in literature^14^,^15^,^16^,^17^,^18^,^19^, allowing us to investigate the thermoelectric effect in the tunnelling regime. To measure the DNA conductance and thermal electric effect, we modified T base at the 3′ end with an amino group, which binds to gold electrodes to establish electrical contact between the electrodes and the molecule^20^.

### Conductance

We measured the DNA conductance and thermoelectric effect with a temperature controlled scanning tunnelling microscopy (STM) set-up shown in Fig. 1a, following a procedure described in detail elsewhere^21^ (see also the Methods section). The experiment generated conductance versus STM tip–substrate distance traces, showing plateaus associated with the formation and breakdown of single-molecule junctions (marked with arrows in the red traces in Fig. 1c). As a control experiment, we performed the experiment without DNA and observed no characteristic plateaus in the conductance traces (black traces). By collecting thousands of conductance traces, we constructed a conductance histogram (Fig. 1d), which shows a broad peak, where the peak position measures the average conductance of a single dsDNA molecule. The width of the histogram peak is not due to experimental error, but rather it reflects the intrinsic variability and distribution in the molecule–electrode binding geometry^22^. To determine the experimental error, we repeated the above experiment more than three times, each using a new sample to generate thousands of conductance traces, for every DNA sequence, and used the s.d. in the conductance values as the error bar.

We characterized the DNA samples by performing non-denaturing polyacrylamide gel electrophoresis, circular dichroism spectroscopy and melting temperature experiments in phosphate buffer (Supplementary Figs 1, 2 and 3; Supplementary Methods 1, 2 and 3). These experiments confirmed that each DNA sequence studied here was in B-form double-helical structure in phosphate buffer. To ensure the DNA molecule retained its double-helical structure in the experiments (∼20% relative humidity), we compared the conductance of DNA measured in humidified air with that measured in phosphate buffer and found similar values within the experimental uncertainty (Supplementary Figs 4 and 5). Another parameter that characterizes molecular junction stability is the stretching length, which is the average distance that one can stretch the junction before it breaks. We also found similar stretching lengths measured in phosphate buffer and in humidified air (Supplementary Fig. 6; Supplementary Discussion 1). These facts led us to conclude that the DNA molecules in humidified air retained its double-helical structure, possibly due to a thin absorbed water layer on the substrate surface. This conclusion is further supported by atomic force microscopy^23^, STM^24^, mechanical break junction^25^ and polarization modulation infrared^26^ measurements of dsDNA on a surface in the air.

**Figure 2:**
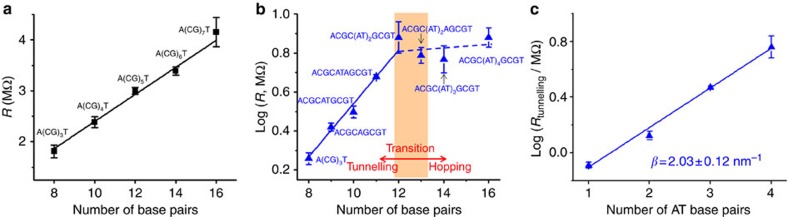
DNA resistance versus molecular length. (**a**) A(CG)_*n*_T (*n*=3–7), (**b**) ACGC(**AT**)_*m*_GCGT (m=0–4) and ACGC(**AT**)_*m*−1_**A**GCGT (*m*=1–3) sequences, and (**c**) the AT blocks ((AT)_*m*_ and (AT)_*m*−1_A) in ACGC(AT)_*m*_GCGT and ACGC(AT)_*m*−1_AGCGT sequences. The solid lines in **a**,**b** and **c** are linear fits to the data, and the dashed line in **b** is a guide to eye. The transition from tunnelling to hopping occurs the AT block is longer than 4 base pairs, which is markers with a shaded orange region.

The conductance (or resistance) of DNA depends on both the molecular length and sequence. For A(CG)_*n*_T (*n*=3–7), the resistance increases linearly with the molecular length (Fig. 2a), which is expected for hopping transport^20^,^27^,^28^. According to the hopping model^29^,^30^, holes hop along a DNA molecule from one end to another, where the individual G bases act as hopping sites such as stepping stones. Consequently, the total resistance is proportional to the number of hopping sites (G bases), and thus the length of the molecule (see more discussions in Supplementary Fig. 7 and Supplementary Discussion 2). When DNA is connected to two electrodes (tip and substrate), an additional contribution to the resistance arises from the two contacts. The total resistance of the DNA is given by^29^,^30^


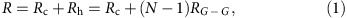


where *R*_c_ is contact resistance, *R*_*h*_ is intrinsic DNA resistance, *N* is number of base pairs and *R*_*G−G*_ is resistance of a hopping step (one GC base pair). Table 2 summarizes *R*_c_ and *R*_h_ of all the A(CG)_*n*_T sequences. By fitting the experimental data with equation (1), we found that the contact resistance, *R*_c_=0.48±0.16 MΩ, and the resistance of one step hopping, *R*_*G−G*_=0.28±0.02 MΩ per GC base pair. Note that the contact resistance is small compared with the total resistance for A(CG)_*n*_T (see Table 2), indicating efficient electronic coupling between the electrodes and the molecule, and the DNA resistance is dominated by the molecule itself, rather by the contact.

In contrast to A(CG)_*n*_T described above, the resistance length dependence of ACGC(**AT**)_*m*_GCGT/ACGC(**AT**)_*m*−1_**A**GCGT is significantly different (Fig. 2b). For *m*=0, 1 and 2, the resistance increases more rapidly with length (*L*) and can be best fitted with an exponential function, rather than with a shallow linear dependence found for A(CG)_*n*_T. For *m*>2, however, the resistance becomes weakly length dependent, indicating a transition in the charge transport mechanism at *m*=2 (or 4 A–T base pairs). This observation agrees with previous photochemical and direct conductance measurements of DNA, which have shown that inserting a short AT block in the middle of a GC sequence introduces a tunnelling barrier, leading to an exponential increase in the DNA resistance with the AT block length^14^,^15^,^16^,^17^,^18^,^19^, and inserting a longer AT block results in the transition in the charge transport mechanism from tunnelling to hopping^14^,^16^. The tunnelling–hopping transition has also been predicted by theoretical calculations^14^,^15^,^16^,^17^,^18^,^19^. See Supplementary Discussion 2 for further discussion.

On the basis of the above results, we modelled the ACGC(**AT**)_*m*_GCGT and ACGC(**AT**)_*m*−1_**A**GCGT resistance as a sum of three contributions, electrode–DNA contact, hopping via GC sequences and tunnelling via the AT block, given by *R*=*R*_c_+*R*_h_+*R*_t_, where *R*_c_ is the contact resistance, *R*_h_ is the GC hopping resistance and *R*_t_ is the AT tunnelling resistance. Since both *R*_c_ and *R*_h_ are known from the A(CG)_*n*_T measurement, the above relation allowed us to determine the resistance of the inserted AT block, which is listed in Table 2. Semi-logarithmic plot of the AT block resistance (*R*_t_) versus the AT block length shows an exponential dependence, following *R*_t_=*R*_0_exp(*βL*), where *R*_0_ is the contact resistance between the GC and AT block, and *β* is the decay constant and *L* is the AT block length (Fig. 2c). Fitting the experimental data with the above exponential relation leads to a *β* of 2.03±0.12 nm^−1^ (Fig. 2c). This decay constant is smaller than ∼4 nm^−1^ reported by Xu *et al*.^15^ for thiolated DNA molecules, but is within the range reported by various other works^8^,^9^,^10^,^11^,^12^,^13^ and consistent with the calculations by Torisi *et al*.^19^ and Voityuk *et al*.^31^ for dsDNA with alternating AT sequences.

### Thermoelectric effect

The thermoelectric effect of a single DNA–electrode junction can be expressed by


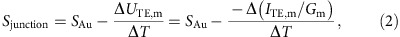


where *S*_Au_ is the Seebeck coefficient of gold (∼2 μV K^−1^ (ref. 32)), *U*_TE,m_ is the open-circuit voltage, *I*_TE,m_ is the short-circuit current, *G*_m_ is the conductance of the molecular junction and Δ*T* is the temperature difference between the STM tip and substrate electrodes. We measured the thermoelectric effect by holding the tip at 295 K while cooling the substrate from 295 to 275 K (ref. 33). The selection of this temperature range ensures that all the dsDNA sequences are structurally stable, and their resistance does not change significantly with temperature^34^.

**Figure 3:**
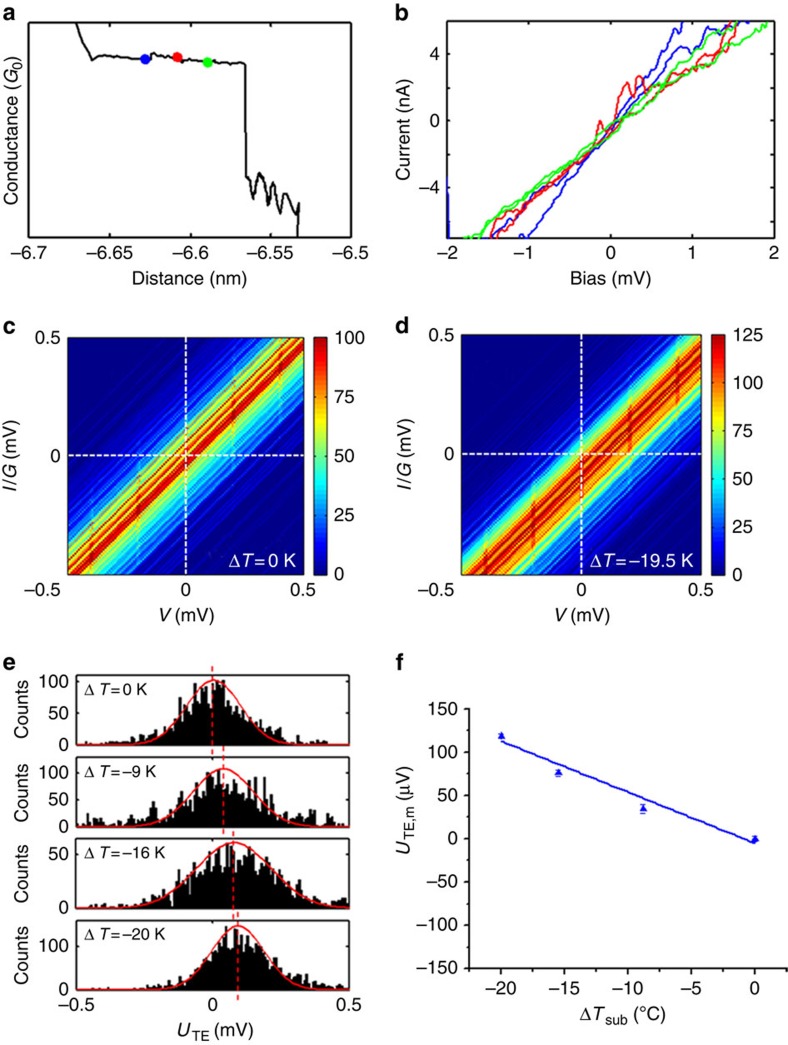
Thermoelectric measurement of DNA. (**a**) Semi-logarithmic plot of conductance–distance trace (the coloured dots mark, where *I–V* curves are measured). (**b**) Individual *I–V* curves measured at the locations marked with corresponding colour in **a**. (**c**) *I/G–V* histogram at Δ*T*=0 K. (**d**) *I/G–V* histogram at Δ*T*=19.5 K, showing an offset due to the thermoelectric effect. (**e**) Thermal voltage histogram, where the red curves show Gaussian fits. (**f**) Thermoelectric voltage versus Δ*T*, where the straight line is linear fit to the data, and error bars are fitting errors. DNA sequence: ACGC(**AT**)_2_GCGT.

The thermoelectric effect measurement started by detecting plateaus in the conductance traces during the retraction of the STM tip from the substrate (Fig. 3a). Once a plateau was detected, the tip retraction was halted and the bias voltage between the tip and substrate electrodes was swept over ±10 mV to obtain a current (*I*)–voltage (*V*) characteristic curve. After recording an *I*–*V* curve, the tip was further retracted to a new position, and the *I*–*V* measurement was repeated until the molecular junction broke down. Figure 3b shows several *I*–*V* curves for ACGC(**AT**)_2_GCGT measured at different locations of a conductance plateau, where the colours of curves match the colours of the dots marked on the conductance plateau shown in Fig. 3a. These *I*–*V* curves are linear within the bias range.

From the *I*–*V* characteristics, we obtained the average Seebeck coefficient of a DNA molecule with the following procedure. First, each *I*–*V* curve was fitted with a linear function to obtain *G*, and then a corresponding *I*/*G*–*V* curve was obtained by dividing *I* by *G*. From thousands of *I*/*G*–*V* curves, a two-dimensional *I*/*G*–*V* histogram was constructed for each temperature difference (Fig. 3c,d). When the substrate is held at the same temperature as the tip (room temperature), the centre of the histogram passes through the origin of *I*/*G*–*V* plot (Fig. 3c). However, when the substrate is cooled below the tip temperature, there is an offset in the histogram in the *I*/*G*–*V* plot, due to the thermoelectric effect (Fig. 3d). By extracting the voltage offsets (at zero current) from the *I*/*G*–*V* histograms, we constructed the thermoelectric voltage histograms at different tip–substrate temperature differences (Fig. 3e; Supplementary Figs 8 and 9; Supplementary Table 1; Supplementary Discussion 3). The peak in each thermoelectric voltage histogram was fitted with a Gaussian distribution, from which *U*_TE,m_ was determined. Figure 3f shows that *U*_TE,m_ depends linearly on Δ*T*, and the slope was used to determine *S*_junction_, the Seebeck coefficient of the dsDNA molecule according to equation (2).

**Figure 4:**
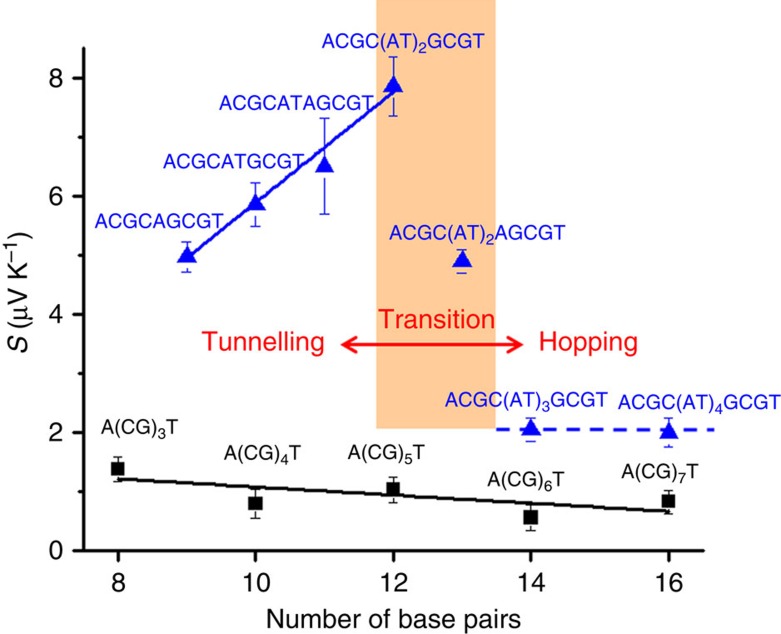
Seebeck coefficients of DNA with different molecular lengths and sequences. The black squares are the Seebeck coefficients of A(CG)_*n*_T (*n*=1, 2, 3). The blue triangles are the Seebeck coefficients of ACGC(**AT**)_*m*_GCGT (*m*=1–4) and ACGC(**AT**)_*m*−1_**A**GCGT (*m*=1−3) sequences, showing a transition when the AT block length is longer than 4 base pairs. The solid and dashed lines are guides to eye.

Figure 4 summarizes the Seebeck coefficients of DNA with different lengths and sequences, from which we can draw several important conclusions. First, the Seebeck coefficients are positive for all the DNA sequences in both the tunnelling and hopping regimes studied here. It has been predicted that a positive Seebeck coefficient corresponds to hole-dominated charge transport and a negative Seebeck coefficient signals electron-dominated charge transport^2^. The observation of positive Seebeck coefficients here indicates that holes dominate charge transport in DNA. This is expected because the DNA HOMO level is close to the electrode Fermi level compared with its LUMO level, and is also consistent with the previous experiments^14^,^16^,^18^,^19^,^35^. However, the prediction of the Seebeck coefficient sign is based on a coherent tunnelling model, which is not necessarily applicable to hopping transport. The data shown in this indicate that this prediction appears to be valid also in the hopping regime. Second, the Seebeck coefficients of DNA in the hopping regime (in A(CG)_*n*_T) are small, and weakly depend on the molecular length compared with other organic molecules^36^,^37^. Last, inserting a short AT block (shorter than 5 AT base pairs) into the middle of A(CG)_*n*_T leads to a much greater Seebeck coefficient, and it increases with the AT block length. However, when the AT block is longer than 5 AT base pairs, the Seebeck coefficient drops to the level of A(CG)_*n*_T and become insensitive the AT length. This transition coincides with the observed tunnelling–hopping transition near 4–5 AT base pairs, which strongly suggests that the thermoelectric effect is large in the tunnelling regime and small in the hopping regime. We discuss these observations below.

For bulk materials, the Seebeck coefficient can be expressed by the Mott formula^38^,


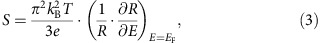


where *R* is the energy-dependent resistance function and *E* is the energy of the carriers. *k*_B_ is the Boltzmann constant, *T* is temperature and *e* is elementary charge. The Mott formula assumes incoherent transport, which is applicable to hopping transport in DNA. For coherent tunnelling transport, the Seebeck coefficient can be obtained with Landauer's formula, which leads to a similar expression as equation (3) except that *R* is replaced by the energy-dependent transmission probability function^5^,^39^.

**Figure 5:**
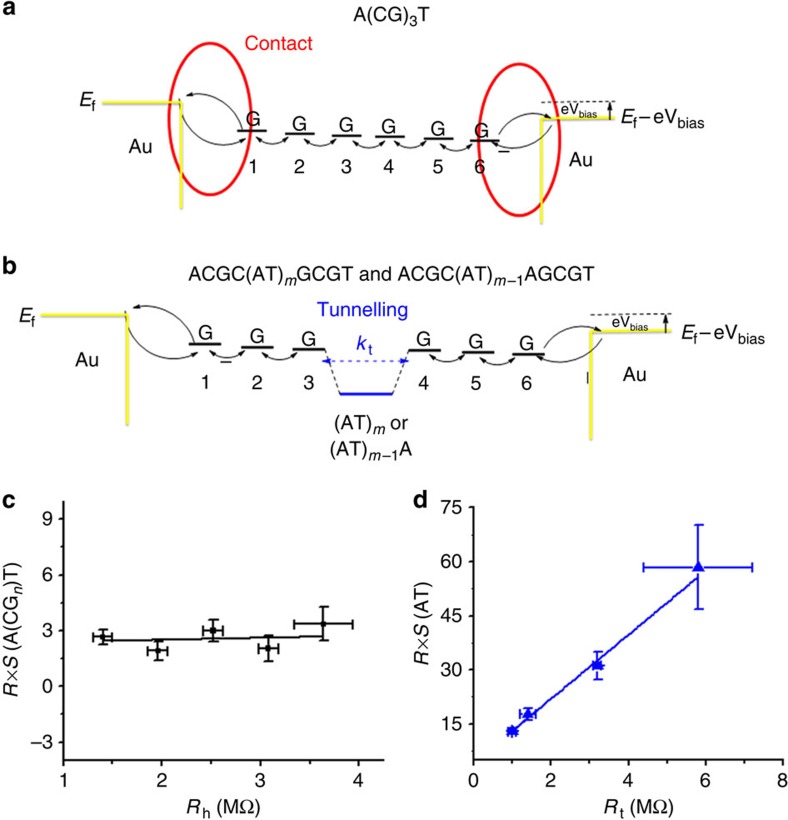
Thermoelectric effect in DNA in terms of tunnelling and hopping models. (**a**) Charge transport in A(CG)_*n*_T, where a hole is injected from left electrode into the first G, then hops along the molecule with each G as a hopping site, and eventually reaches the right electrode. The charge transfer rates at the contacts (red part) are energy dependent, which is a dominant contribution to the Seebeck coefficient. (**b**) Charge transport in ACGC(**AT**)_*m*_GCGT and ACGC(**AT**)_*m*−1_**A**GCGT, where a tunnelling barrier (marked blue) arises from the AT block. (**c**) *R* × *S* versus *R*_h_ for A(CG)_*n*_T sequences. (**d**) *R* × *S* versus *R*_t_ for ACGC(**AT**)_*m*_GCGT and ACGC(**AT**)_*m*−1_**A**GCGT (*m*=1 and 2). Solid lines in **c** and **d** are linear fits to the data.

We analyse the experimental thermoelectric effect in the hopping regime with equation (3) by assuming that the resistance function, *R(E)*, can be expressed as a sum of the contact and hopping resistance functions (equation (1)), which is modelled as Fig. 5a. Consequently, the overall Seebeck coefficient (*S*) consists of contributions from the molecule–electrode contact (*S*_c_) and hopping along the dsDNA sequences (*S*_h_), given by


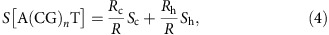


where *R*, *R*_c_ and *R*_h_ in equation (4) are the total resistance, contact resistance and DNA resistance, respectively. We discuss the validity of the assumption and derivation of equation (4) in Supplementary Discussion 4. Both *R*_c_ and *R*_h_ are known from the linear fitting to the experimental data shown in Fig. 2a (Table 2).

In the case of ACGC(**AT**)_*m*_GCGT and ACGC(**AT**)_*m*−1_**A**GCGT sequences, the total resistance *R*[AT] must include a contribution, *R*_t_, arising from the inserted AT block in the middle of the GC sequence, as shown in Fig. 5b. The overall Seebeck coefficient of the DNA junction must also include the contribution from the inserted AT block, expressed by,


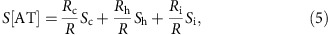


where *S*_t_ is the Seebeck coefficient of the inserted AT part.

In equation (4), although *R*_h_ is proportional to the molecular length, *S*_h_ is independent of the length because it is proportional to the relative change in *R*_h_ (a normalized quantity). *S*_c_ is also independent of the molecular length. So, we conclude that the molecular length dependence of the overall Seebeck coefficient, S, arises from *R* in equation (4), which as shown in Fig. 2a is weakly dependent on molecular length. By fitting the measured Seebeck coefficients times *R* with *R*_h_ at different lengths (Fig. 5c), we obtained the slope, *S*_h_=0.1±0.3 μV K^−1^, and from the intercept we found that *S*_c_=4.8±1.7 μV K^−1^, showing that the contact plays a dominant role in the thermoelectric effect of A(CG)_*n*_T. The above model explains the weak length dependence of the observed thermoelectric effect.

For equation (5), we plotted *S* × *R* versus *R*_t_ (Table 2) in Fig. 5d for short AT blocks (*m*=1–2), which follows a linear function. According to equation (5), the slope of the linear relation is *S*_t_, from which we found that *S*_t_=8.9±1.4 μV K^−1^. For longer AT blocks (*m*>2), we determined *S*_t_ from equation (5) (Table 3). Compared with the short AT blocks, these sequences have smaller Seebeck coefficients (∼2 μV K^−1^).

To further understand the tunnelling Seebeck coefficient, *S*_t_, from the AT block, we express the transmission function of the tunnelling barrier with a Lorentzian distribution by^33^,^36^,^37^,^40^


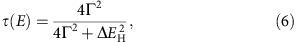


where Γ is the width of the HOMO level of the AT block and Δ*E*_H_ is the energy difference between the AT block and GC HOMO levels. Using the above transmission function, the contributions of the AT block to the resistance and Seebeck coefficient are expressed as


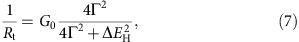



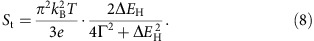


By fitting the experimental data with equations (7) and (8), we obtained Γ and Δ*E*_H_ (Table 3), which shows that Γ<<Δ*E*_H_, indicating a weak coupling between the AT block and the GC sequences and electrodes. In this weak-coupling limit, *S*_t_ is proportional to 1/Δ*E*_H_ (refs 33, 41). Because the electronic coupling between alternating AT base pairs in the AT block is weak^31^, Δ*E*_H_ is expected to be weakly dependent on the AT block length, which explains the constant slope (*S*_t_) in Fig. 5d.

The above hopping and tunnelling models help understand the length dependence of the Seebeck coefficients in DNA. However, to explain that the insertion of a short AT block increases the Seebeck coefficient, a model including sufficient chemical and structural details of dsDNA is needed. We used an extended Su–Schrieffer–Heeger model with the Lennard–Jones potential to describe the dsDNA (see Supplementary Fig. 10 and Supplementary Discussion 5 for details). The theory reproduces sequence dependence, and also the observed large increase in the Seebeck coefficient associated with the insertion of an AT block in the middle of CG sequences (Supplementary Fig. 11). The increase can be attributed to the difference in the site energies between AT and GC base pairs. However, we emphasized that the calculation was performed at 8 K. As such, the theory cannot provide quantitative agreement with the experimental Seebeck coefficient nor it predicts the transition from tunnelling to hopping. Further investigation is thus still needed to quantitatively describe the measured Seebeck coefficients and the tunnelling–hopping transition.

In this paper we, have studied the charge transport and thermoelectric effect in single DNA molecules with different sequences and lengths. The transport mechanism in CG sequences (5′-A(CG)_*n*_T-3′, with *n*=3–7) is hole hopping, as reflected by linear dependence of the resistance on the molecular length. The Seebeck coefficient that describes the thermoelectric effect is small and weakly dependent on the molecular length. Inserting a block of AT base pairs in the middle of a CG sequence (ACGC(**AT**)_*m*_GCGT-3′, *m*=1–4; and 5′-ACGC(**AT**)_*m*−1_**A**GCGT-3′, *m*=1–3) changes both the charge transport and thermoelectric effect substantially. When the inserted AT block is shorter than 4 base pairs, it acts as a tunnelling barrier with resistance increases exponentially with the block length, and its Seebeck coefficient is large compared with the CG sequences. However, when the inserted AT block is longer than 4 base pairs, the charge transport mechanism changes from tunnelling to hopping, the resistance becomes weakly length dependent and the Seebeck coefficient drops to smaller values. The experimental results have been analysed in terms on tunnelling and hopping models, and compared with an extended Su–Schrieffer–Heeger model with the Lennard–Jones potential. The work demonstrates that the DNA thermoelectricity may be tuned by its length and sequence, and studying the thermoelectric effect provides new insights into charge transport in DNA.

## Methods

### Preparation of dsDNA SAM

DNA molecules with different sequences and lengths were purchased from Bio-Synthesis Inc. The T terminal of each DNA molecule was modified with an amine group^20^, allowing binding to gold electrodes for electrical measurement. The name and structure of all the sequences are listed in Table 1. The DNA molecules were annealed using a thermal cycler (TC-050-18, Labnet Inc) to form the B-form double-helical structure in phosphate buffer, which was verified by gel electrophoresis, circular dichroism and melting curve (Supplementary Figs 1, 2 and 3; Supplementary Methods 1, 2 and 3). The phosphate buffer was prepared with Na_2_HPO_4_.2H_2_O (for high-performance liquid chromatography (HPLC) ≥98.5%) and NaH_2_PO_4_ (for HPLC ≥99%) purchased from Fluka. The DNA sample was immobilized on a gold substrate (gold with thickness of ∼160 nm on mica) prepared in a vapour deposition system under ultrahigh vacuum. Before each experiment, the gold substrate was annealed with hydrogen flame briefly, and immediately immersed in phosphate buffer (5 mM, pH=7) containing 5 μM double-helical DNA, incubated overnight. The substrate electrode was then rinsed with phosphate buffer and dried with nitrogen gas. The DNA-coated gold substrate was attached to the sample holder of the STM, which was placed in a glove box under nitrogen atmosphere (relative humidity ∼20%).

### STM set-up

The charge transport and thermoelectric measurements were carried out with the STM consisting of a controller (Nanoscope IIIA, Digital Instruments Inc.) and a STM scanner (Molecular Imaging). The STM tip was freshly cut from a gold wire (0.25 mm diameter, 99.95%, Alfa Aesar). A piece of electrically shielded copper strip (0.7 × 0.4 × 2 cm) was thermally connected to the STM tip holder to serve as a thermal reservoir. The STM sample holder was mounted on a semiconductor cooler with a thermistor to measure the temperature (controlled by Lake Shore 331 temperature controller).

### Measurement of thermoelectricity

The STM break junction technique was used to measure the conductance of DNA with a procedure described in detail elsewhere^21^ (Fig. 1a). Briefly, the STM tip was first brought into contact with the DNA molecules on the gold substrate, allowing the amine-terminated DNA to bind with the tip and substrate electrodes. The tip was then retracted from the substrate, during which a conductance–distance trace was recorded. The last plateau in the conductance trace corresponded to a single DNA molecule attached to the tip and substrate electrodes to form a molecular junction. The process was repeated thousands of times. A conductance histogram was constructed from the individual conductance traces, and the peak in the conductance histogram gave the average conductance of the DNA molecule.

To measure the thermoelectric effect of DNA molecules, a temperature gradient was created across the molecule by cooling the sample holder while maintaining the STM tip at the room temperature^33^. The temperature of the sample was monitored with thermistors during the experiment. When a molecular junction was formed in the STM break junction measurement as indicated by the formation of a plateau feature in the conductance–distance trace, the tip was held in position and an *I*–*V* curve was recorded. The tip was then retracted further away from the substrate by an additional distance. If the current did not drop abruptly, signalling that the molecular junction was still intact, another *I*–*V* curve was recorded. Otherwise, the measurement was started over again.

Thousands of *I*–*V* curves were collected for each temperature gradient. These *I*–*V* curves were normalized by conductance (*G*) and presented as a two-dimensional *I*/*G* versus *V* histogram. The voltage offsets in the *I*/*G* versus *V* histogram were used to construct a histogram of the thermoelectric effect, and Gaussian fitting of the thermoelectric effect histogram provided the Seebeck coefficient of DNA. Controlling the STM tip movement, recording and processing the experimental data described above were achieved with Labview 8.5 and data acquisition card.

## Additional information

**How to cite this article:** Li, Y. *et al*. Thermoelectric effect and its dependence on molecular length and sequence in single DNA molecules. *Nat. Commun.* 7:11294 doi: 10.1038/ncomms11294 (2016).

## Supplementary Material

Supplementary Information Supplementary Figures 1-11, Supplementary Table 1, Supplementary Discussion, Supplementary Methods and Supplementary References

## Figures and Tables

**Table 1:** Sequences of dsDNA studied in this work.

Name	Duplex	Abbreviation
A(CG)_3_T	5′-ACGCGCGT-3′; 3′-TGCGCGCA-5′	A(CG)_*n*_T
A(CG)_4_T	5′-ACGCGCGCGT-3′; 3′-TGCGCGCGCA-5′	A(CG)_*n*_T
A(CG)_5_T	5′-ACGCGCGCGCGT-3′; 3′-TGCGCGCGCGCA-5′	A(CG)_*n*_T
A(CG)_6_T	5′-ACGCGCGCGCGCGT-3′; 3′-TGCGCGCGCGCGCA-5′	A(CG)_*n*_T
A(CG)_7_T	5′-ACGCGCGCGCGCGCGT-3′; 3′-TCGGCGCGCGCGCGCA-5′	A(CG)_*n*_T
ACGC**A**GCGT	5′-ACGCAGCGT-3′; 3′-TGCGTCGCA-5′	ACGC(AT)_*m*−1_AGCGT
ACGC**AT**GCGT	5′-ACGCATGCGT-3′; 3′-TGCGTACGCA-5′	ACGC(AT)_m_GCGT
ACGC**ATA**GCGT	5′-ACGCATAGCGT-3′; 3′-TGCGTATCGCA-5′	ACGC(AT)_*m*−1_AGCGT
ACGC(**AT**)_2_GCGT	5′-ACGCATATGCGT-3′; 3′-TGCGTATACGCA-5′	ACGC(AT)_*m*_GCGT
ACGC(**AT**)_2_**A**GCGT	5′-ACGCATATAGCGT-3′; 3′-TGCGTATATCGCA-5′	ACGC(AT)_*m*−1_AGCGT
ACGC(**AT**)_3_GCGT	5′-ACGCATATATGCGT-3′; 3′-TGCGTATATACGCA-5′	ACGC(AT)_*m*_GCGT
ACGC(**AT**)_4_GCGT	5′-ACGCATATATATGCGT-3′; 3′-TGCGTATATATACGCA-5′	ACGC(AT)_*m*_GCGT

dsDNA, double-stranded DNA.

**Table 2:** Conductance and Seebeck coefficients of DNA with different sequences.

Sequence	Conductance peak position (log(*G*/*G*_0_))*	Conductance peak width	Resistance (MΩ)	*R*_c_ (MΩ)†	*R*_h_ (MΩ)†	*R*_t_ (MΩ)†	Seebeck coefficient (μV K^−1^)
A(CG)_3_T	−2.17±0.03	0.20	1.9±0.1	0.48	1.40	NA	1.4±0.2
A(CG)_4_T	−2.27±0.02	0.18	2.4±0.1	0.48	1.96	NA	0.8±0.2
A(CG)_5_T	−2.37±0.01	0.17	3.0±0.1	0.48	2.52	NA	1.0±0.2
A(CG)_6_T	−2.42±0.01	0.18	3.4±0.1	0.48	3.08	NA	0.6±0.2
A(CG)_7_T	−2.51±0.03	0.20	4.2±0.3	0.48	3.64	NA	0.8±0.2
ACGC**A**GCGT	−2.31±0.02	0.25	2.6±0.1	0.48	1.12	1.0	5.0±0.3
ACGC**AT**GCGT	−2.36±0.03	0.35	3.0±0.2	0.48	1.12	1.4	5.9±0.4
ACGC**ATA**GCGT	−2.57±0.01	0.30	4.8±0.1	0.48	1.12	3.2	6.5±0.8
ACGC(**AT**)_2_GCGT	−2.76±0.08	0.30	7.4±1.4	0.48	1.12	5.8	7.9±0.5
ACGC(**AT**)_2_**A**GCGT	−2.68±0.04	0.50	6.1±0.6	0.48	1.12	4.5	4.9±0.2
ACGC(**AT**)_3_GCGT	−2.65±0.07	0.52	5.8±0.9	0.48	1.12	4.2	2.0±0.2
ACGC(**AT**)_4_GCGT	−2.77±0.05	0.40	7.6±0.9	0.48	1.12	6.0	2.0±0.3

NA, not applicable.

^*^G_0_ (=2*e*^2^/*h*=77.48 μS) is the conductance quantum.

^†^*R*_c_, *R*_h_ and *R*_t_ are the contact resistance, hopping part resistance (CG sequences) and tunnelling part (AT sequences) resistance, respectively.

**Table 3:** The resistance (*R*_t_) and the Seebeck coefficients (*S*_t_) of the AT blocks, from which the broadening of the HOMO levels (Γ), and the energy difference between the HOMO levels of AT and GC blocks were determined.

Sequences	*R*_t_ (MΩ)	*S*_t_ (μV K^−1^)	Γ (eV)	Δ*E*_H_ (eV)
ACGC**A**GCGT	1.0±0.3	8.9±1.4	0.07±0.02	1.3±0.4
ACGC**AT**GCGT	1.4±0.4	8.9±1.4	0.06 ±0.02	1.3±0.4
ACGC**ATA**GCGT	3.2±0.3	8.9±1.4	0.05±0.01	1.6±0.3
ACGC(**AT**)_2_GCGT	5.8±1.6	8.9±1.4	0.03±0.01	1.4±0.5
ACGC(**AT**)_2_**A**GCGT	4.5±0.8	6.1±1.3	NA	NA
ACGC(**AT**)_3_GCGT	4.2±1.1	2.2±0.8	NA	NA
ACGC(**AT**)_4_GCGT	6.0±1.1	2.1±0.7	NA	NA

HOMO, highest occupied molecular orbital; NA, not applicable.
