# Supplementary material for: Thermoelectric effect and its dependence on molecular length and sequence in single DNA molecules
Source: Nat Commun. 2016 Apr 15;7:11294. doi: 10.1038/ncomms11294 (PMC4835548; doi:10.1038/ncomms11294)
Supplement: Supplementary Information — Supplementary Figures 1-11, Supplementary Table 1, Supplementary Discussion, Supplementary Methods and Supplementary References [file ncomms11294-s1.pdf]

Supplementary Figures

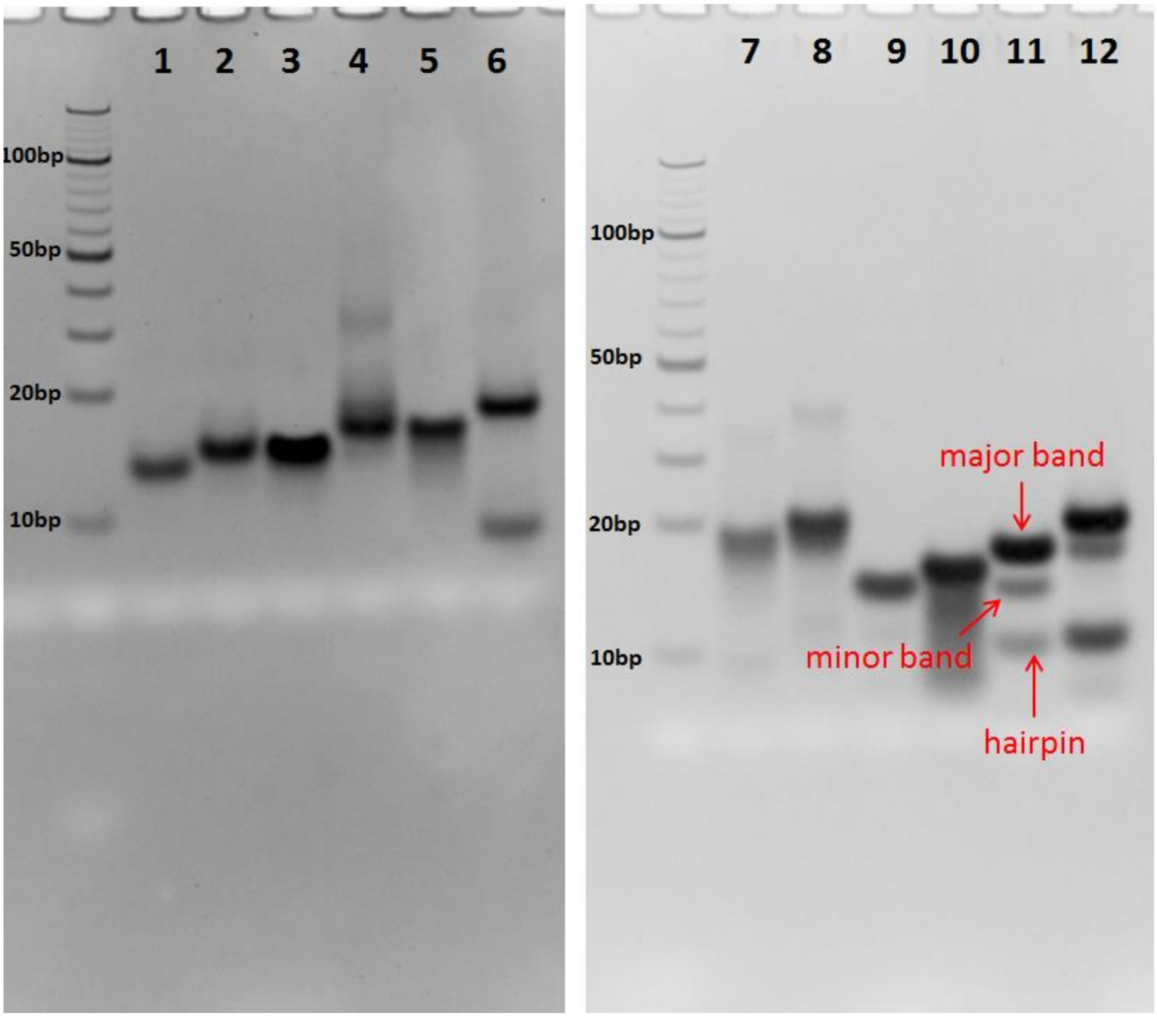

Supplementary Figure 1. Gel electrophoresis of dsDNA samples after a programed annealing process. The samples from 1 to 12 are A(CG)$_3$T, A(CG)$_4$T, ACGC**AT**GCGT, A(CG)$_5$T, ACGC(**AT**)$_2$GCGT, ACGC(**AT**)$_3$GCGT, A(CG)$_6$T, A(CG)$_7$T, ACGC**A**GCGT, ACGC**ATA**GCGT, ACGC(**AT**)$_2$**A**GCGT, and ACGC(**AT**)$_4$GCGT with their complementary sequences, respectively. Weak bands due to hairpin formation and protected amine linker appear in ACGC(**AT**)$_3$GCGT, ACGC(**AT**)$_2$**A**GCGT, and ACGC(**AT**)$_4$GCGT.

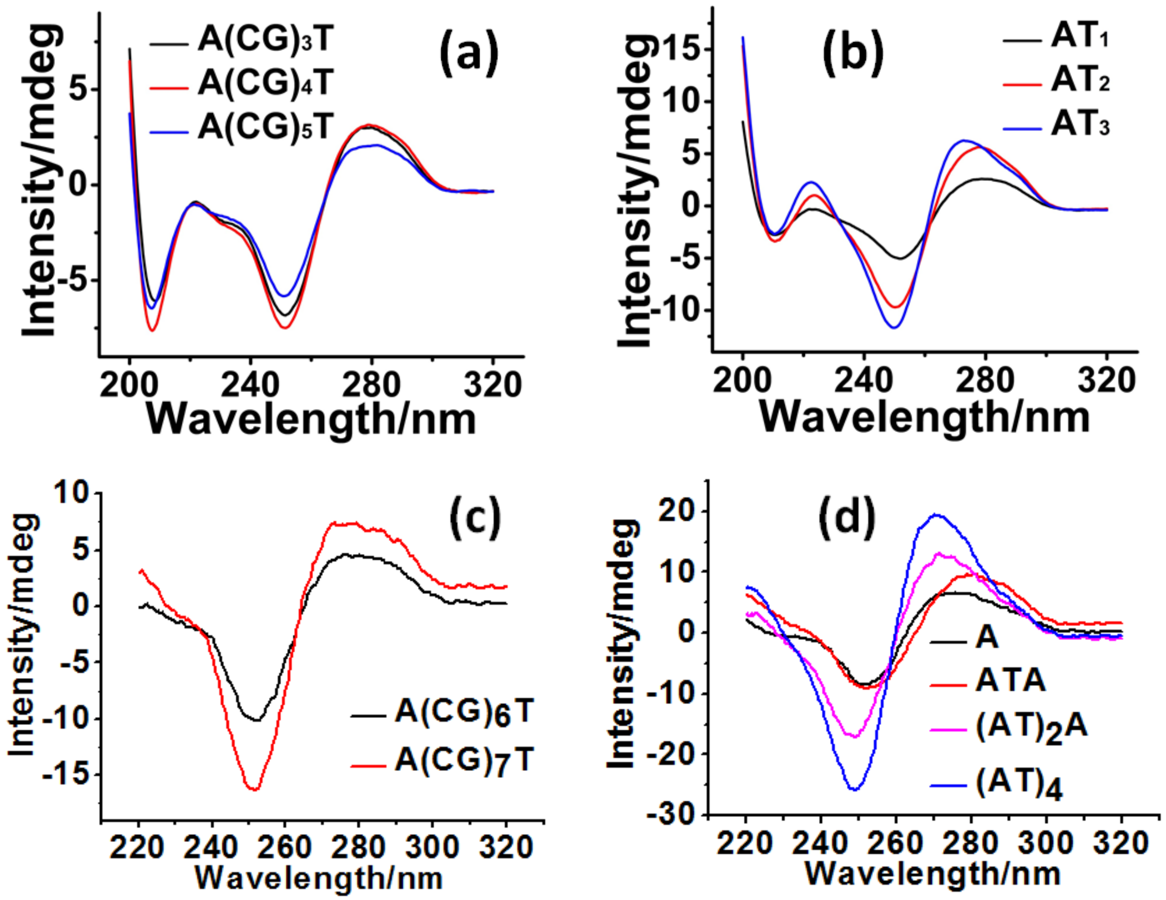

Supplementary Figure 2. Circular dichroism data of **(a)** A(CG)$_n$T (n=3~5), **(b)** ACGC(AT)$_m$GCGT (m=1~3), **(c)** A(CG)$_n$T (n=6,7), **(d)** ACGC(**AT**)$_{m-1}$**A**GCGT (m=1~3) and ACGC(**AT**)$_m$GCGT (m=4).

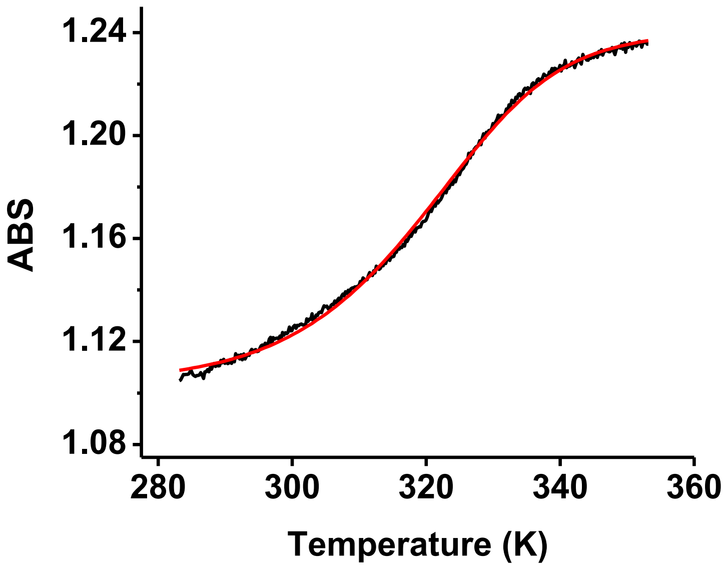

Supplementary Figure 3. Melting temperature absorption curve for A(CG)$_3$T (black curve), and two-state thermodynamic model fitting (red curve).

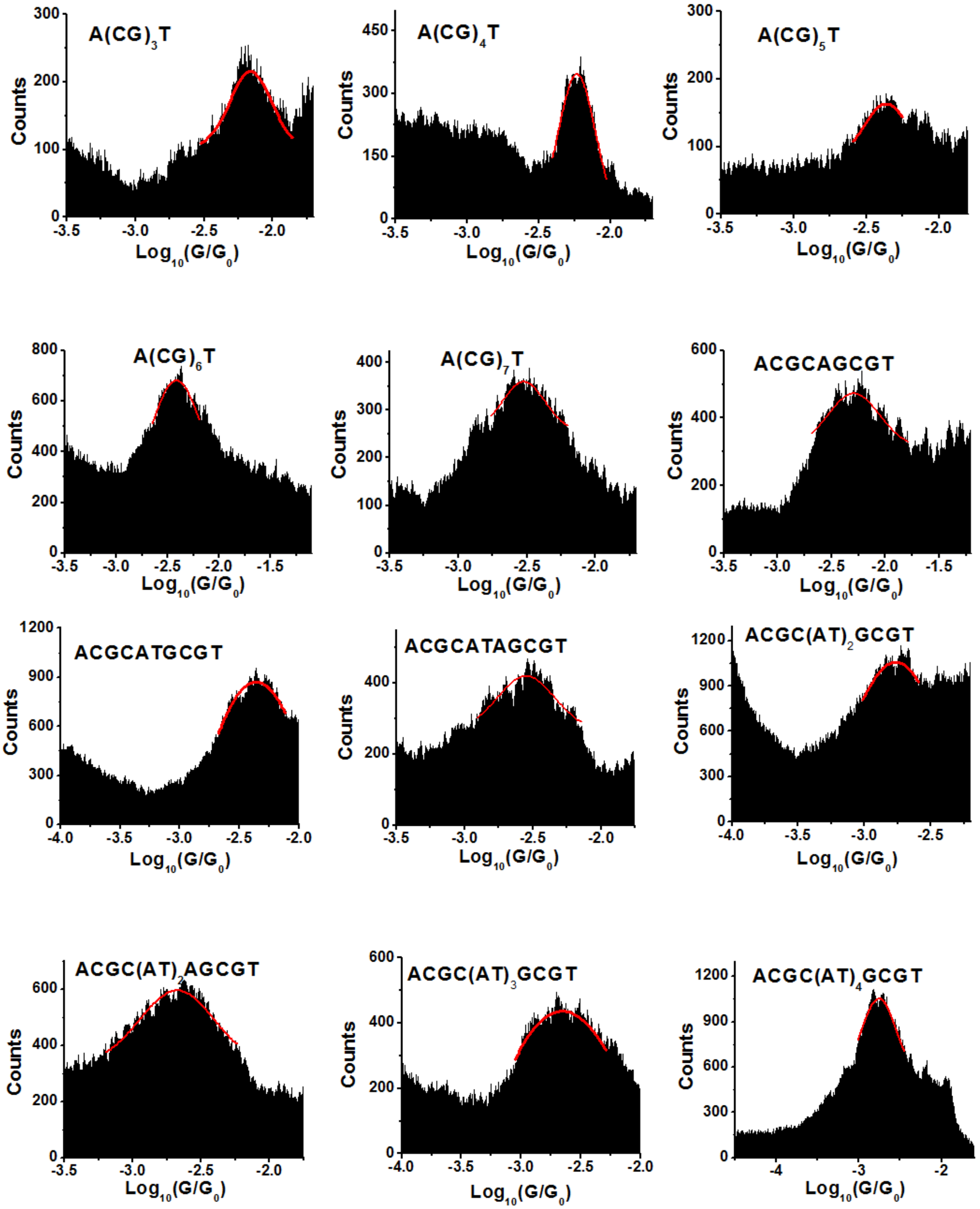

Supplementary Figure 4 Conductance histograms of dsDNA measured in humidified air. Red curves are Gaussian fittings to the conductance peaks.

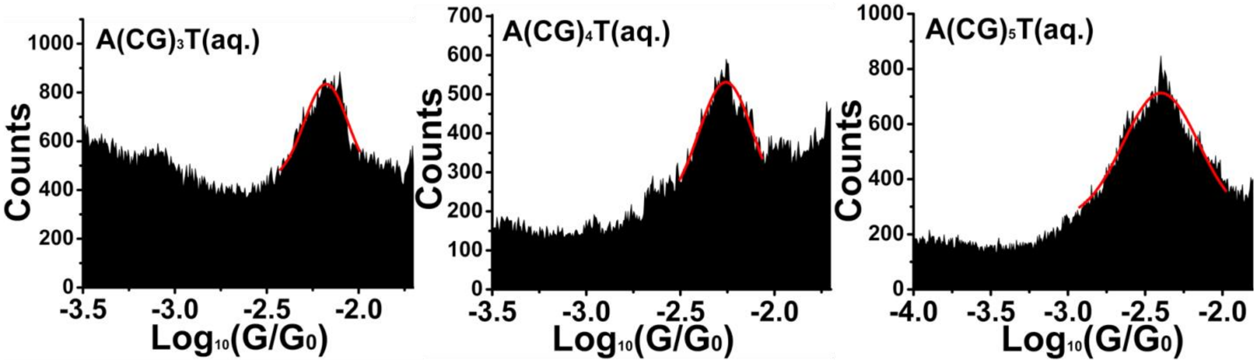

Supplementary Figure 5 Conductance histograms of dsDNA measured in solution. Red curves are Gaussian fittings of the conductance peaks.

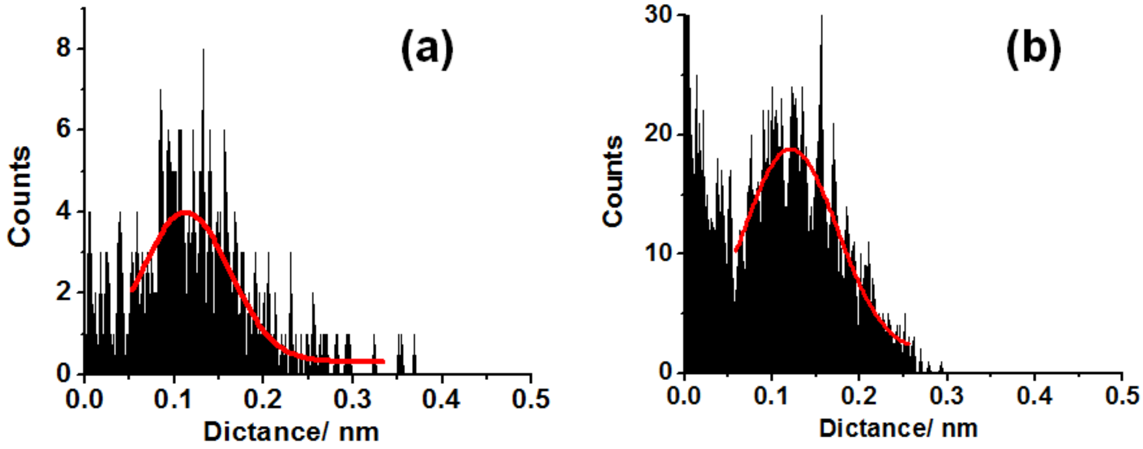

Supplementary Figure 6. Step-length histograms of A(CG)$_3$T measured (a) in the air and (b) in solution. The red curves show Gaussian fittings of the histogram peaks.

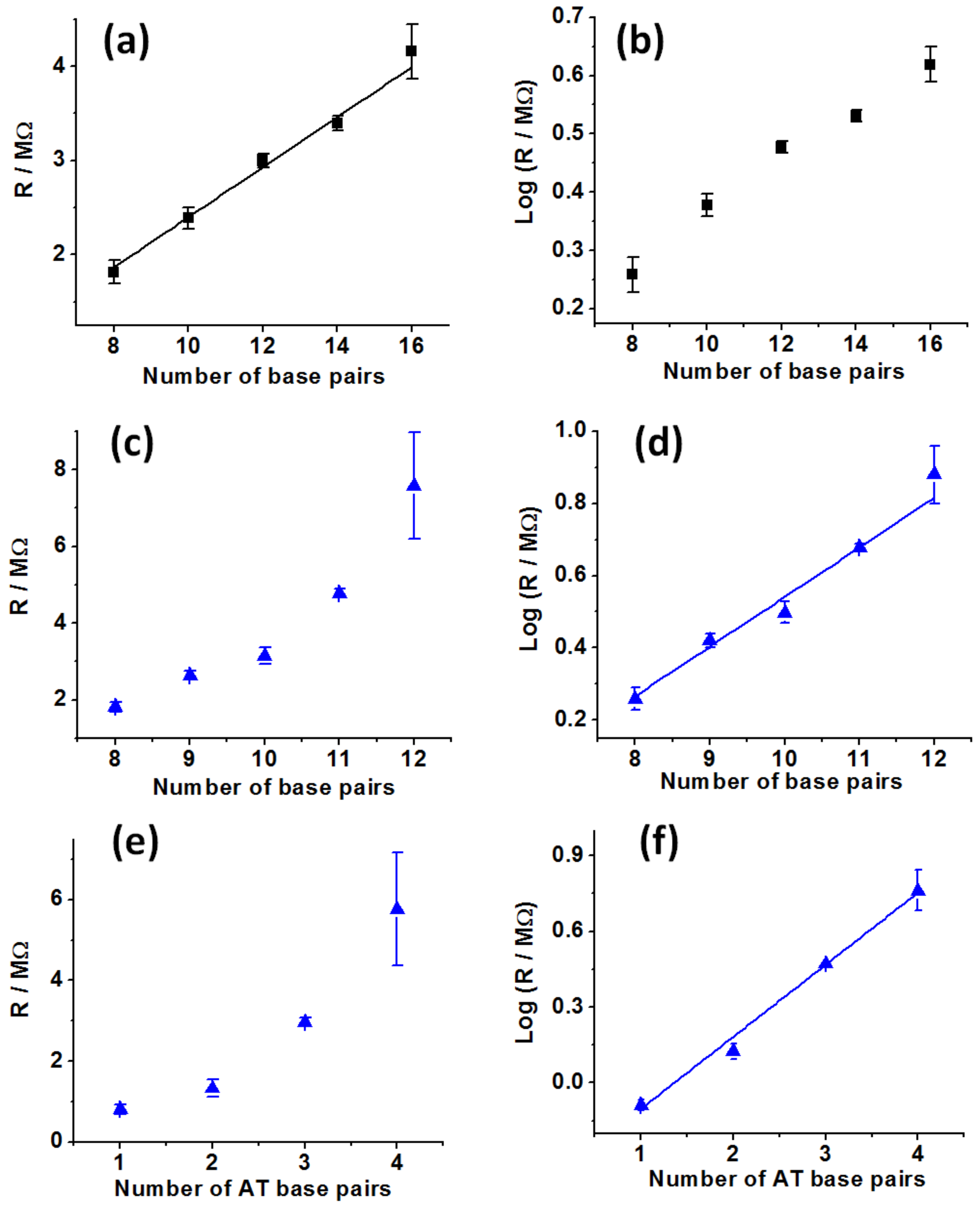

Supplementary Figure 7 Linear and semi-logarithmic plots of resistance vs. length of different DNA sequences. Linear (a) and semi-logarithmic (b) plots of resistance vs. length for A(CG)$_n$T (n=3~7). Linear (c) and semi-logarithmic (d) plots of resistance vs.

length for ACGC$(AT)_m$GCGT (m=1~4) and ACGC$(AT)_{m-1}$AGCGT (m=1~3). Linear (e) and semi-logarithmic (f) plots of resistance vs. length for the inserted AT blocks in ACGC$(AT)_m$GCGT (m=1~4) and ACGC$(AT)_{m-1}$AGCGT (m=1~3).

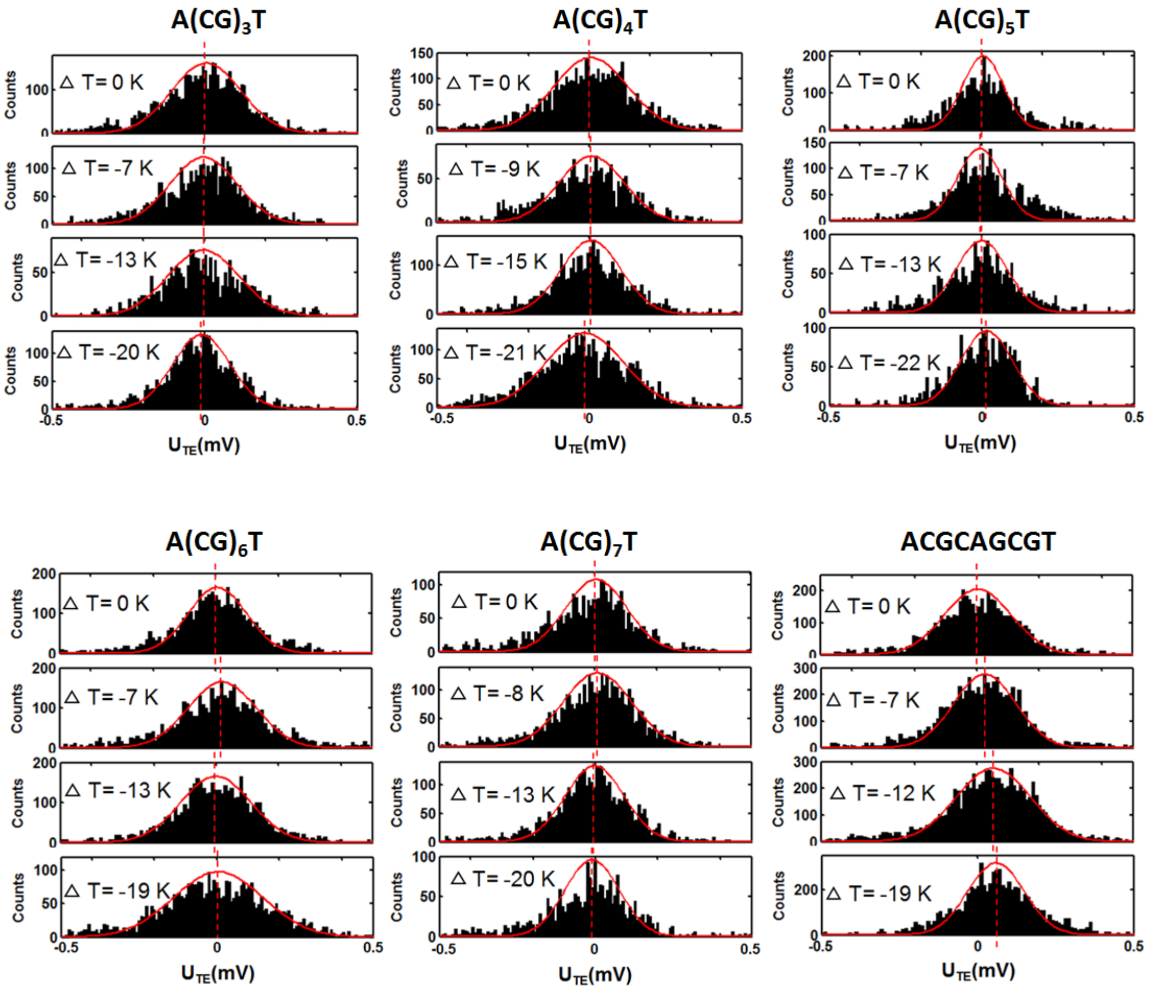

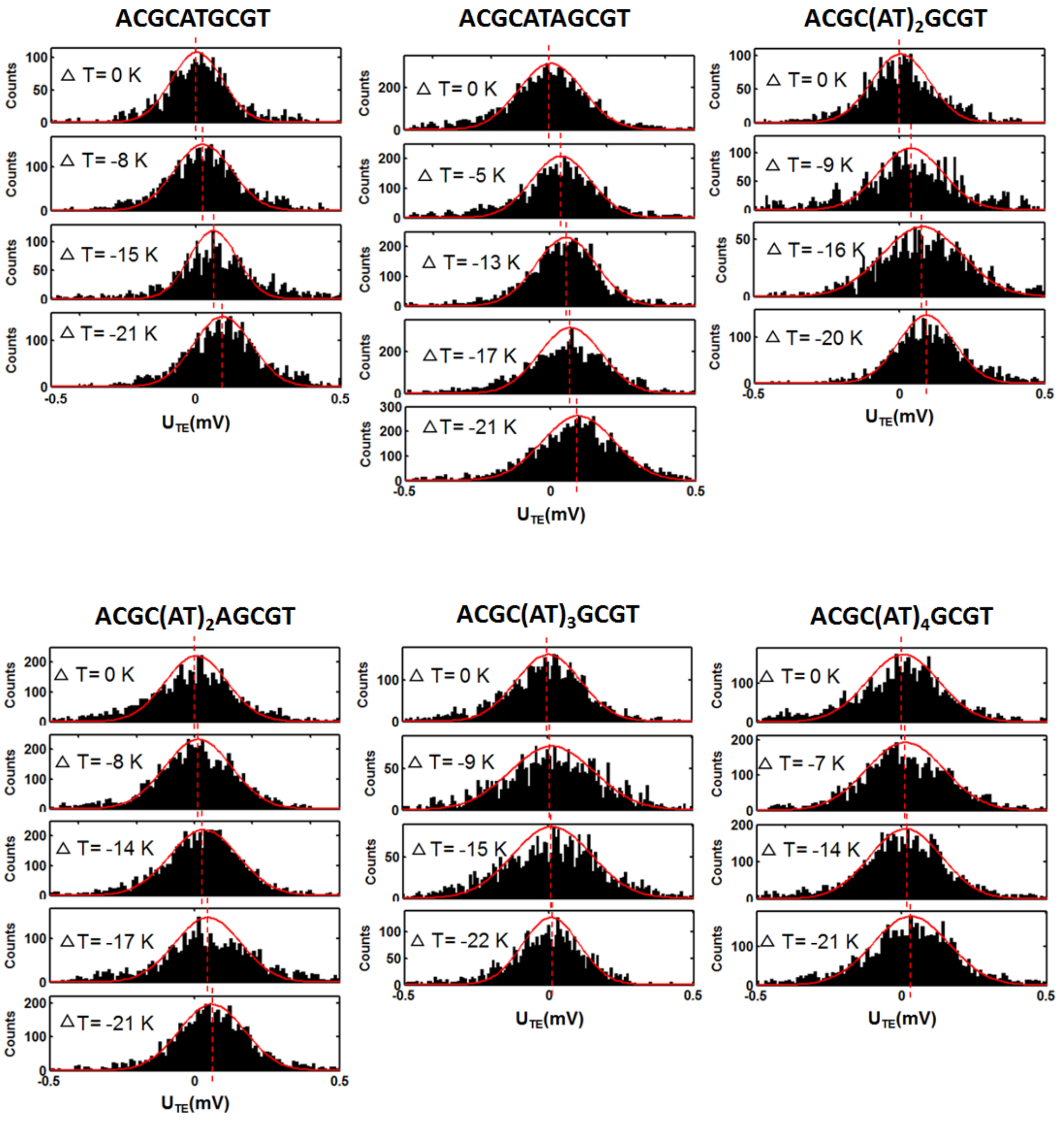

Supplementary Figure 8 Thermal voltage histograms for double helical DNA collected at different temperature gradients. The red curves are Gaussian fitting of the voltage histogram peaks, and the dash lines indicate the peak positions.

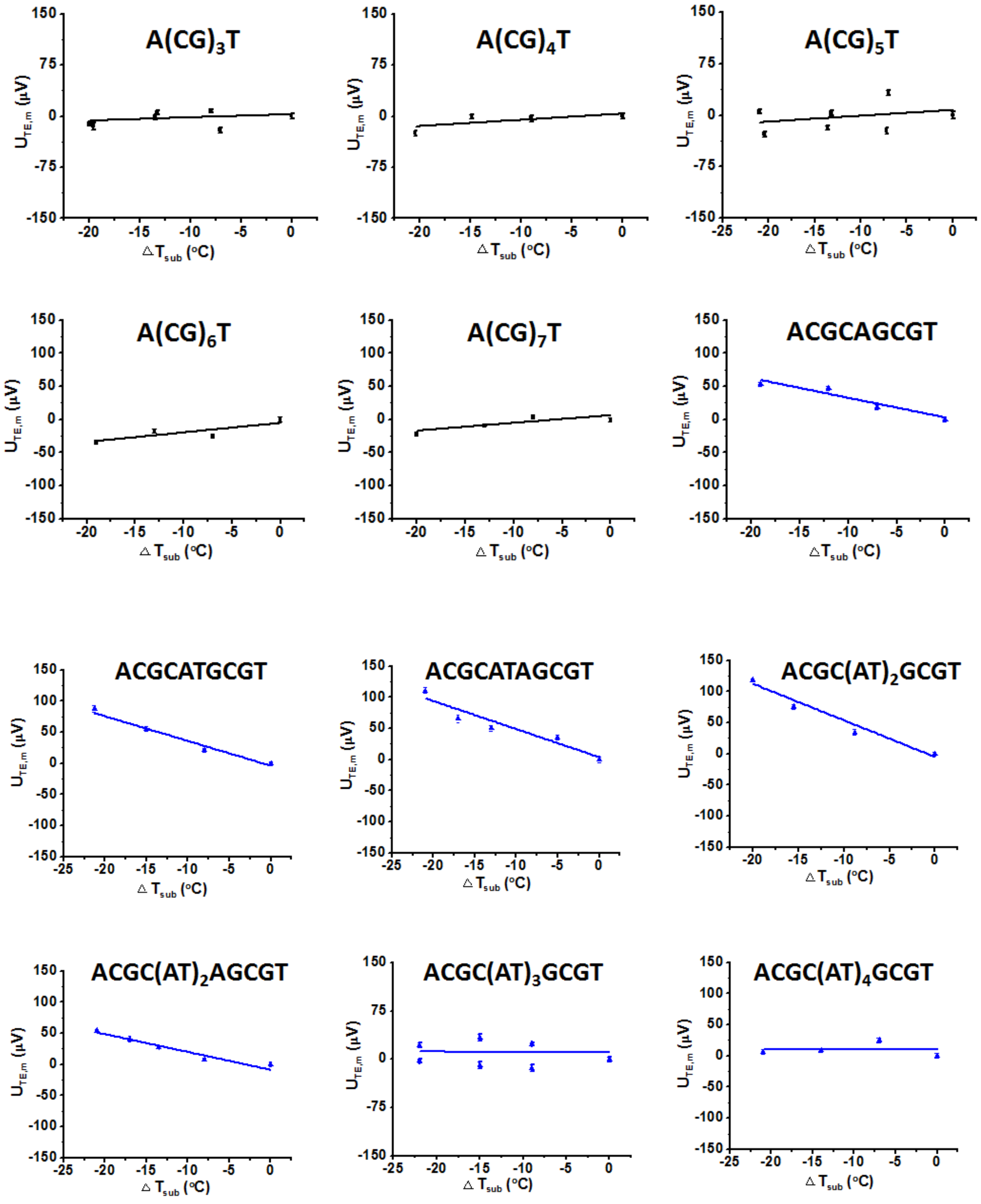

Supplementary Figure 9 Linear fitting of the thermoelectric voltage data at different temperature gradients for $A(CG)_nT$ (n=3~7), $ACGC(AT)_mGCGT$ (m=1~4), and $ACGC(AT)_{m-1}AGCGT$ (m=1~3) sequences.

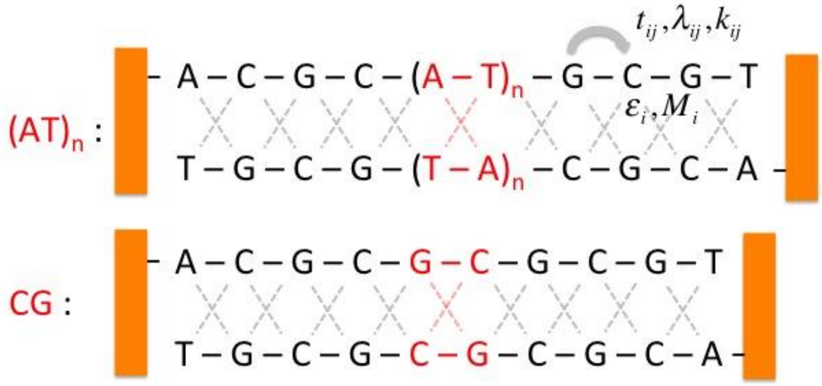

$$H_{SSH+LJ} = -\sum_{\langle ij \rangle, \sigma} t_{ij}\left[1-\lambda_{ij}\left(u_i - u_j\right)\right]\left(c_{i\sigma}^{\dagger} c_{j\sigma} + H.c.\right) + \sum_{i,\sigma} \varepsilon_i n_{i\sigma}$$

$$+1/2\sum_{j=1}^{N} M_j\left(du_j/dt\right)^2 + 1/2\sum_{ij}^{N} u_i \Phi\left(\vec{R}_i - \vec{R}_j\right) u_j$$

Supplementary Figure 10. The Su-Schrieffer-Heeger model of dsDNA, where ε_i and M_i are the site energy and mass, respectively, t_{ij} is the transfer integral, λ_{ij} is describes the electron-phonon coupling, and k_{ij} is force field constant.

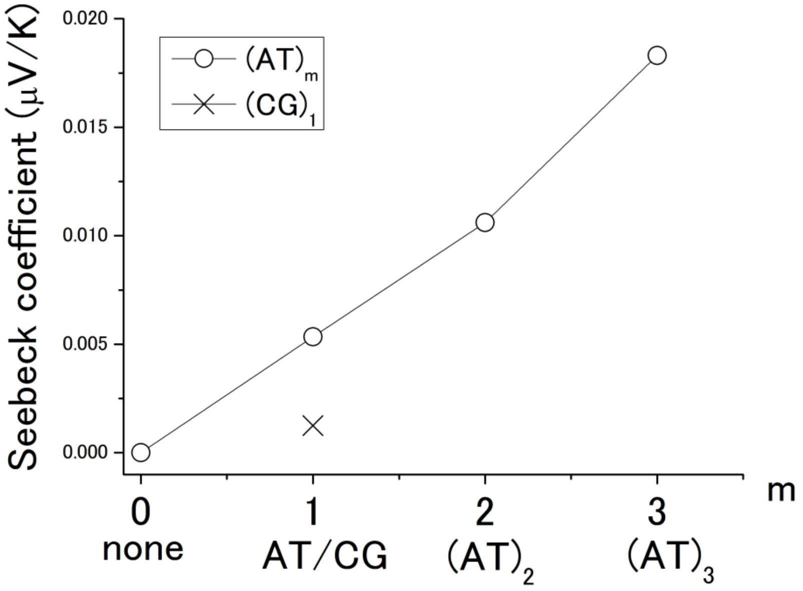

Supplementary Figure 11. Seebeck coefficient and its dependence on DNA sequence and length. The Seebeck coefficients are for the inserted part in the middle of sequence. AT, $(AT)_2$, $(AT)_3$, CG and none means calculation for one, two, three alternating AT units, one CG alternating unit, and no molecule.

Supplementary Table

Supplementary Table 1 FWHM of thermal voltage histograms

Sequence	FWHM1 (μV)	FWHM2 (μV)	FWHM3 (μV)	FWHM4 (μV)	FWHM5 (μV)	mean (μV)	standard deviation (μV)
$A(CG)_3T$	283	286	278	241		272	21
$A(CG)_4T$	289	274	261	298		281	16
$A(CG)_5T$	193	201	235	233		215	22
$A(CG)_6T$	240	273	279	322		279	34
$A(CG)_7T$	268	280	244	239		258	20
ACGCAGCGT	276	251	289	238		264	23
ACGCATGCGT	242	263	235	264		251	15
ACGCATAGCGT	274	268	272	279	296	278	11
$ACGC(AT)_2GCGT$	253	280	303	247		271	26
$ACGC(AT)_2AGCGT$	279	288	294	295	285	288	7
$ACGC(AT)_3GCGT$	293	327	316	249		296	35
$ACGC(AT)_4GCGT$	295	314	297	300		301	9

* From FWHM1 to FWHM5, the data is acquired from increasing temperature gradient.

Supplementary Discussion

1 Stretching length of dsDNA in the air and in solution

The stretching length is the distance over which a DNA junction can be stretched before breakdown in the STM break junction experiment, which reflects the breakdown of the hydrogen bonds at the very end of the dsDNA. Supplementary Fig.4 shows the stretching length histograms of $A(CG)_3T$ in the air and in solution, and Gaussian fittings to the histograms. From the fittings, the average stretching lengths were found to be 0.11 nm and 0.12 nm, respectively. The similarity in the stretching length measured in air and in solution confirms that the dsDNA retained its native structure in humidified air.

2. Molecular length dependence of DNA resistance

The resistance of $A(CG)_nT$ sequences is weakly length dependent, which can be fitted with a linear relation (Supplementary Figure 7a). Plotting the same data in a logarithmic scale shows a roughly linear behavior with the reception of the first data point (Supplementary Figure 7b). This is not surprising because an exponential function, $R=R_0\exp(-\beta L)$, can be expressed as linear as R~R0(1- βL), for small βL. However, forcing the fit of data with the exponential function leads to $\beta=0.29\pm0.02$ nm^{-1}, which is small. This result is consistent with the previously reported STM break-junction[1] and photochemistry data[2,3].

Compared to $A(CG)_nT$, the resistance of $ACGC(AT)_mGCGT$ and $ACGC(AT)_{m-1}AGCGT$ is more strongly length dependent (see Fig. 2b in main text). The insertion of the AT blocks in the middle of the GC sequences introduce a large change in the length dependence of the DNA resistance. The resistance of the sequences in linear scale shows large deviation from the simple linear length dependence (Supplementary Figure 7c).

Note that only the data for the AT blocks shorter than 4 base pairs are plotted here. If the resistance for the sequences with longer AT blocks were included, the deviation from the linear dependence is even larger. In contrast to the linear scale plot, the semi-logarithmic plot shows a more linear behavior (Supplementary Figure 7d), indicating exponential length dependence of the DNA resistance. From the slope of the semi-logarithmic plot, we determined that $\beta = 1.20 \pm 0.12$ nm^{-1}, far larger than that for $A(CG)_nT$. This observation supports that the AT blocks act as tunneling barriers to give rise to exponential length dependence of the resistance.

ACGC$(AT)_m$GCGT and ACGC$(AT)_{m-1}$AGCGT contains GC blocks, which are expected to act as hopping sites. In other words, tunneling in these sequences arises only from the AT blocks. We thus analyzed the data by excluding the resistance contribution from the GC blocks. The linear scale plot shows large deviation from the simple linear length dependence (Supplementary Figure 7e). In contrast, the semi-logarithmic plot (Supplementary Figure 7f) shows good linear dependence, confirming the tunneling behavior, and the exponential decay constant, β, was found to be 2.03 ± 0.12 nm^{-1}, which is similar to those in other molecular wire system[4]. This observation also agrees with the theoretical predictions[5-7] and experimental results[2,8] reported in literature.

3. Thermoelectricity measurements

Thermoelectric voltage histograms measured at different temperature gradients for all the sequences are presented in Supplementary Fig. 8. The peak positions determined from Gaussian fitting (with the error bars determined from the fitting errors) were plotted against the temperature gradient and fitted with a linear relation (Supplementary Fig. 9).

From the slope of the fitted line, the Seebeck coefficient of the DNA molecule was obtained based on Eq. 2. The variations in U_{TE} are originated from the variations in single molecular conductance, rather from experimental errors[9,10]. We have listed the fwhms for all the sequences in the supporting information, which shows 10-15% standard deviations (Supplementary table 1). Despite the variability in fwhm, we cannot identify a systematic dependence of the fwhm on DNA sequence.

4. Derivations of Eq. 4 in the main text

If we assume that the energy dependent resistance function, R(E), of $A(CG)_nT$, is the sum of contact (R_c) and hopping (R_h) contributions, we can express the Seebeck coefficients of $A(CG)_nT$ sequences as

$$S\left[A(CG)_nT\right]=\frac{\pi^2k_B^2T}{3e}\cdot\left[\left(\frac{1}{R}\cdot\frac{\partial R_c}{\partial E}\right)_{E=E_F}+\left(\frac{1}{R}\cdot\frac{\partial R_h}{\partial E}\right)_{E=E_F}\right]. \qquad (1)$$

Supplementary Eq. 1 can be simplified as,

$$S\left[A(CG)_nT\right]=\frac{\pi^2k_B^2T}{3e}\cdot\frac{R_c}{R}\cdot\left(\frac{1}{R_c}\cdot\frac{\partial R_c}{\partial E}\right)_{E=E_F}+\frac{\pi^2k_B^2T}{3e}\cdot\frac{R_h}{R}\cdot\left(\frac{1}{R_h}\cdot\frac{\partial R_h}{\partial E}\right)_{E=E_F} \quad \text{(Eq. 4 in main text)}$$

$$=\frac{R_c}{R}S_c+\frac{R_h}{R}S_h$$

, leading to Eq. 4 in the main text. Eq. 6 in the main text can also be derived from this.

Alternatively, we may assume that the total thermal voltage as a sum of the contact and hopping thermal voltages, given by [11]

$$\Delta V=S_c\Delta T_c+S_h\Delta T_h \quad (2)$$

If we further assume that total temperature difference is a sum of the contact and hopping temperature differences, or [11]

$$\Delta T = \Delta T_c + \Delta T_h \quad (3).$$

Finally, we assume that heat transports along from left to right electrodes along the molecule without loss sideway, or

$$\Delta T_{h,c} = (\kappa / \kappa_{h,c})\, \Delta T \ (4)$$

, where κ, κ_c and κ_h are the total, contact and hopping thermal conductance. Combing Supplementary Eqs. 2, 3 and 4, we have

$$S = (\kappa / \kappa_c) S_c + (\kappa / \kappa_h) S_h \quad (5)$$

Note that in addition to the above assumptions, Supplementary Eq. 5 consumes that we could define site temperature along a molecule. The validity of the concept is discussed in literature [12,13].

Eq. 4 in the main text and Supplementary Eq. 5 express the total Seebeck coefficient as resistance and thermal resistance (inverse of thermal conductance) weighted sum, respectively. According to the Wiedeman-Franz law, the electrical conductivity is proportional to the thermal conductivity, so Eq. 4 in the main text and Supplementary Eq. 5 are equivalent. The Wiedeman-Franz law is not yet well established in molecular junctions, but one of us[14] showed that the law holds when the energy gap between the electrode Fermi energy and molecular site energy is sufficiently large (compared to the transfer integral). This result was obtained in the tunneling regime. In the hopping region, experimental and theoretical evidence showed that the hopping

region is connected adiabatically to the tunneling region[15], indicating that the Wiedeman-Franz law might be applicable also in the hopping regime, although a complete theory is still lacking.

5. Tight binding theory of DNA thermoelectricity

A theory that treats electron-phonon coupling in the strong coupling regime has been developed to calculate DNA conductance[16]. The theory has been recently extended to determine the Seebeck coefficient in conducting polymers[17]. While the strong coupling theory is useful in predicting qualitative trends of molecular conductance and thermoelectric effect, it cannot include chemical details in the calculations easily. In this respect, the weak coupling theory is more suitable. Previously, the weak coupling theory has been used to explain the tunneling-hopping transition in single oligomers,[18] and to determine other charge transport properties, such as the electric current noise in molecular junctions[19]. In this work, we applied the weak coupling theory to examine the length and sequence dependence of the DNA Seebeck coefficient.

We adopted an extended Su-Schrieffer-Heeger model with the Lennard-Jones potential to describe dsDNA. The model considers electrons, phonons and electron-phonon coupling (See Supplementary Figure 10 for the Hamiltonian). To perform the calculation, we assumed each DNA base as a site with key parameters depicted in Supplementary Figure 10. The parameters that describe the electrons in DNA were determined from the DNA hole transfer rates measured by transient absorption spectroscopy.[20] The phonons in DNA were treated with the nearest-neighbor interaction approximation using the Lennard-Jones potential. The force field between the nearest-neighbor bases was uniform with a force field constant, $k_{ij} = 0.25\, eV/\text{Å}^2$. The mass of

each site was taken to be molecular mass of the base. The electrodes were considered by assuming their Fermi energy to be $E_F = -7.75\ eV$. The electron-phonon coupling constant, $\lambda_{ij} = -0.6$ and temperature, T= 8 K, were used to stabilize the self-consistent calculation. Because the inelastic contribution to the electron-phonon coupling is expected to scale with λ^2, we do not expect that the result change qualitatively for larger λ. The calculated Seebeck coefficient and its dependence on the DNA length and sequence are summarized in Supplementary Figure 11. The calculation shows that the insertion of AT blocks in the middle of GC sequences lead to larger Seebeck coefficients, in agreement with the experimental data. However, the calculation was performed at 8 K, which could not predict the tunneling-hopping transition observed at room temperature. Further work will be needed to fully describe both the conductance and thermoelectricity in DNA.

Supplementary Methods

1. Gel electrophoretic characterization of dsDNA

Nondenaturing polyacrylamide gel electrophoresis was performed to confirm the formation of double helical DNA molecules. In the electrophoretic measurement, a voltage of 200 V was applied to 50 pmol of each sample with 10% nondenaturing PAGE gels in 1×TAE Mg^{2+} buffer at 22 ℃ for 3 hrs. The resulting ethidium bromide (EB) stained gels were scanned in a Biorad Gel Doc XR+ system for visualization. DNA samples included 5'-A(CG)$_n$T-3' (n=3~7), 5'-ACGC(**AT**)$_m$GCGT-3' (m=1~4), and 5'-ACGC(**AT**)$_{m-1}$**A**GCGT-3' (m=1~3) sequences. These DNA samples were dissolved in

phosphate buffer (5 mM, pH=7). The annealing program consisted of holding the temperature at 95 ℃ for 5 mins, cooling from 95 ℃ to 90 ℃ at the rate of 4 ^{o}C per second, cooling from 90 ℃ to 76 ℃ at a rate of 1 ℃ per 5 mins, further cooling from 76 ℃ to 26 ℃ at a rate of 1 ℃ per 15 mins, holding at 25 ℃ for 30 mins, then maintained at 4 ℃. This stepwise annealing process helped prevent the formation of ssDNA hairpin structures. Supplementary Fig. 1 summarizes the electrophoresis gel data for all sequences, which shows one major band for each sequence. For longer sequences, ACGC(**AT**)$_3$GCGT, ACGC(**AT**)$_2$**A**GCGT, and ACGC(**AT**)$_4$GCGT, the electrophoresis indicated a small amount of hairpin structures in the sample. These hairpin structures were formed from single DNA strands, consisting of only one amine linker in each structure, and could not form molecular junctions in the STM break junction experiments. The electrophoresis also revealed a minor band close to the major band of ACGC(**AT**)$_2$**A**GCGT (and ACGC(**AT**)$_4$GCGT), which was due to the protected amine group in some DNA molecules could not be deprotected (Glen Research http://www.glenresearch.com/ProductFiles/Product.php?item=10-1037). Molecules with protected amine linkers could not form molecular junctions in the STM break junction experiments.

2. Circular dichroism (CD) measurements

CD measurements were carried out on Jasco (Easton, MD) J-1710 Spectropolarimeter for 10 uM dsDNA in phosphate buffer (5 mM phosphate buffer, pH=7). Data shown in Supplementary Fig. 2 were average over five scans, collected from 320 nm to 200 nm or 220 nm with a scanning rate of 100 nm min^{-1}. The positive band at around 275 nm and

negative band at around 245 nm indicate a typical B-form structure for all the DNA sequences.

3. Melting temperature

Melting temperature experiment was carried out in a Varian Cary 300 Bio UV spectrophotometer with a Peltier thermal controller to determine melting temperature. 5-μM dsDNA samples were prepared by annealing in 5 mM sodium phosphate buffer, and then heated at a rate of 0.2 K min^{-1} from 283 K (10 ^{o}C) to 353 K (80 ^{o}C) with the absorbance at 260 nm recorded in 60 s intervals. $A(CG)_3T$ (Supplementary Fig. 3) is the shortest sequence measured in this work, which has the lowest melting temperature. The melting curve (black curve) was fitted to a two-state thermodynamic model (red curve), giving a melting temperature of 311±6 K (38 ^{o}C), well above the experimental temperature of 295 K (22 ^{o}C) for the shortest sequence.

Supplementary references:

1 Xiang, L. *et al.* Intermediate tunnelling–hopping regime in DNA charge transport. *Nat. Chem.* **7**, 221-226 (2015).

2 Giese, B., Amaudrut, J., Koehler, A.-K., Spormann, M. & Wessely, S. Direct observation of hole transfer through DNA by hopping between adenine bases and by tunnelling. *Nature* **412**, 318-320 (2001).

3 Fukui, K. & Tanaka, K. Distance dependence of photoinduced electron transfer in DNA. *Angew. Chem., Int. Ed.* **37**, 158-161 (1998).

4 Metzger, R. M. Unimolecular Electronics. *Chem. Rev.* **115**, 5056-5115 (2015).

5 Lewis, F. D., Zhu, H., Daublain, P., Cohen, B. & Wasielewski, M. R. Hole mobility in DNA a tracts. *Angew. Chem., Int. Ed.* **45**, 7982-7985 (2006).

6 Jortner, J., Bixon, M., Voityuk, A. A. & Rösch, N. Superexchange mediated charge hopping in DNA. *J. Phys. Chem. A* **106**, 7599-7606 (2002).

7 Renaud, N., Berlin, Y. A., Lewis, F. D. & Ratner, M. A. Between superexchange and hopping: an intermediate charge-transfer mechanism in Poly(A)-Poly(T) DNA hairpins. *J. Am. Chem. Soc.* **135**, 3953-3963 (2013).

8 Xu, Zhang, Li & Tao. Direct conductance measurement of sngle DNA molecules in aqueous solution. *Nano Lett.* **4**, 1105-1108 (2004).

9 Malen, J. A. *et al.* The Nature of Transport Variations in Molecular Heterojunction Electronics. *Nano Lett.* **9**, 3406-3412 (2009).

10 Quan, R., Pitler, C. S., Ratner, M. A. & Reuter, M. G. Quantitative Interpretations of Break Junction Conductance Histograms in Molecular Electron Transport. *ACS Nano* **9**, 7704-7713 (2015).

11 Apertet, Y., Ouerdane, H., Goupil, C. & Lecoeur, P. Equivalent parameters for series thermoelectrics. *Energ. Confers. Manage.* **93**, 160-165 (2015).

12 Galperin, M., Nitzan, A. & Ratner, M. A. Heat conduction in molecular transport junctions. *Phys. Rev. B* **75**, 155312 (2007).

13 Asai, Y. Theory of local heating in single molecular bridge junctions. *Phys. Rev. B* **84**, 085436 (2011).

14 Yoshihiro, A. Length and energy gap dependences of thermoelectricity in nanostructured junctions. *J. Phys. Condens. Matter* **25**, 155305 (2013).

15 Lee, S. K. *et al.* Universal Temperature Crossover Behavior of Electrical Conductance in a Single Oligothiophene Molecular Wire. *ACS Nano* **6**, 5078-5082 2012).

16 Asai, Y. Theory of Electric Conductance of DNA Molecule. *J. Phys. Chem. B* **107**, 4647-4652 (2003).

17 Wang, Y., Zhou, J. & Yang, R. Thermoelectric Properties of Molecular Nanowires. J. Phys. Chem. *C* **115** (2011).

18 Asai, Y. Nonequilibrium phonon effects on transport properties through atomic and molecular bridge junctions. *Phys. Rev. B* **78**, 045434 (2008).

19 Asai, Y. Vibronic spectroscopy using current noise. *Phys. Rev. B* **91**, 161402 (2015).

20 Senthilkumar, K. *et al.* Absolute Rates of Hole Transfer in DNA. *J. Am. Chem. Soc.* **127**, 14894-14903 (2005).
